# Investigating the functional capacity of porcine uterine natural killer cells during a porcine reproductive and respiratory syndrome virus infection of pregnant gilts

**DOI:** 10.1186/s13567-025-01623-8

**Published:** 2025-10-07

**Authors:** Spencer Sawyer, Melissa R. Stas, Emil Lagumdzic, Maria Stadler, Heinrich Kreutzmann, Christian Knecht, Marlies Dolezal, Armin Saalmüller, Till Rümenapf, Kerstin H. Mair, Andrea Buzanich-Ladinig

**Affiliations:** 1 Clinical Centre for Population Medicine in Fish, Pig and Poultry, Clinical Department for Farm Animals and Food System Science, University of Veterinary Medicine Vienna, Vienna, Austria; 2 Department of Biological Sciences and Pathobiology, Immunology, University of Veterinary Medicine Vienna, Vienna, Austria; 3 Department of Biological Sciences and Pathobiology, Virology, University of Veterinary Medicine Vienna, Vienna, Austria; 4 Department of Biological Sciences and Pathobiology, Platform for Bioinformatics and Biostatistics, University of Veterinary Medicine Vienna, Vienna, Austria

**Keywords:** PRRSV, uNK cells, endometrium, placenta, CD107a, IFN-γ, perforin, Ki-67

## Abstract

Uterine natural killer (uNK) cells play crucial roles in pregnancy, impacting maternal–fetal immune tolerance, angiogenesis, and immune responses. While extensively studied in humans and mice, porcine uNK cells’ functional properties remain relatively underexplored in both healthy uterine environments, as well as during viral infection. This study examined the functional properties of porcine uNK cells isolated from the maternal endometrium (ME) and fetal placenta (FP) in late gestation, comparing healthy gilts to those experimentally infected with porcine reproductive and respiratory syndrome virus (PRRSV), one of the most economically damaging reproductive viruses of the swine industry. Flow cytometry analyses identified three uNK cell subsets based on NKp46 expression to assess subpopulation frequencies and functional variations. Results showed that PRRSV infection did not significantly alter the total NK cell counts but affected NK cell proliferation. NKp46^high^ subsets showed increased Ki-67 expression in samples from experimentally infected gilts. An upregulation of perforin was observed in NKp46^high^ cells, indicating potential immune responsiveness to PRRSV. CD107a degranulation capacity of the examined NK cells was lower in FP uNK cells, possibly due to the fetal immune environment's immaturity. Despite PRRSV exposure, CD107a expression did not significantly change in uNK cells. PRRSV infection led to a suppression of interferon (IFN)-γ expression in most uNK cell subsets. This study provides a look into a previously unstudied aspect of the immune response to PRRSV infection in the porcine uterus and gives new insights into porcine NK cell subsets in an important non-lymphoid organ.

**Supplementary Information:**

The online version contains supplementary material available at 10.1186/s13567-025-01623-8.

## Introduction

Natural killer (NK) cells are a class of lymphocytes belonging to the group 1 innate lymphoid cells (ILCs), which are characterized by their large and highly granular morphology, and capacity to mediate cytotoxicity independently of prior antigen exposure [1–4]. In humans, around 15% of lymphocytes circulating in the peripheral blood are NK cells, which can be divided into CD56^dim^CD16^bright^ and CD56^bright^CD16^dim^ subsets [5]. These two subpopulations of NK cells exist in the blood in a ratio of approximately 10 to 1 and can be differentiated functionally from one another by the high cytolytic capacity of CD56^dim^CD16^bright^ NK cells and the increased capacity of CD56^bright^CD16^dim^ NK cells to produce cytokines [6]. The most prominent cytokine products of NK cells in general are IFN-γ and tumor necrosis factor α (TNF-α), but they are also known to produce the immune inhibitory cytokine interleukin 10 (IL-10) [7].

In contrast to their minority presence among NK cells in the blood, CD56^bright^CD16^dim^ NK cells are known to be the dominant subtype of NK cells in the human uterus [8]. These uterine NK cells (uNK) have been shown to make up 30% of endometrial lymphocytes in non-gravid human and murine uterus, and as much as 70% during early gestation in the gravid uterus [8, 9]. Establishment of pregnancy and initial placenta formation occur in association with considerable changes to the uterine immune environment, with uNK cells showing the most dramatic changes in population size and functional activity [10]. During the pre-implantation phase of pregnancy, uNK cells have been shown to accumulate around the sites of conceptus attachment [11]. During and following attachment, uNK cells produce large quantities of the pro-angiogenic factors vascular endothelial growth factor (VEGF), placental growth factor (PGR), and IFN-γ, which has been shown to be a critical contribution for successful spiral artery remodeling [12, 13]. Although it has been shown that uNK cells decrease in both absolute numbers and in frequencies relative to other uterine immune cells following the completion of placental formation, their presence persists into the later stages of gestation, suggesting a continued role in pregnancy beyond placental formation [14]. In addition to their role in spiral artery remodeling, uNK cells have also been shown to promote an immunotolerant environment through the production of cytokines such as IFN-γ and IL-10, which is more receptive to the invading trophoblasts through mechanisms such as the inhibition of T_H_17 cells and induction of T regulatory cells [15]. During murine pregnancy, uNK cells have been shown to control T_H_17 cells via an IFN-γ-dependent mechanism in the later stages of pregnancy [16]. Although uNK cells have been clearly associated with establishing an environment of immunotolerance at the maternal–fetal interface, uNK cells have also been shown to play a role in immune response to viral infection including PRRSV in swine and ZIKA in humans [17, 18].

Results on human/mouse uNK cells cannot be directly transferred to pigs, due to their different placentation. The morphological characteristics of the porcine placenta, classified as a diffuse epitheliochorial placenta, allow for separate investigation of the maternal epithelium (ME) and fetal placenta (FP) due to the preservation of all layers of maternal and fetal tissue [19]. The interface of maternal and fetal tissue at the site of attachment maintains clear differentiation of maternal and fetal epithelia despite extensive interdigitation of the two layers [20]. The tight barrier prevents direct fetal trophoblast contact with maternal cells and blocks movement between the maternal endometrium and the fetal placenta of cells or sub-cellular structures such as maternal antibodies [19].

Identifying uNK cells and their function in the porcine uterus presents a unique problem, because unlike human and murine uNK cells, there is no defined phenotype which can be used to readily differentiate between porcine peripheral blood NK cells (pbNK) and uNK cells, or even a pan-marker to exclusively identify NK cells in general. Previous investigations have described the porcine NK cell phenotype as perforin^+^CD2^+^CD3^−^CD4^−^CD5^−^CD6^−^CD8α^+^CD8β^−^ CD11b^+^CD16^+^ [21, 22]. An additional marker available is NKp46, which has been described as the best cross-species NK cell marker in mammals [1, 23]. Porcine NK cells exhibit a spectrum of NKp46 expression, including NKp46^−^, which can be used to identify porcine NK cells belonging to different functional groups [24, 25]. In our previous work, we were able to use the phenotype perforin^+^CD3^+^CD8α^+^CD16^+^ to identify a subpopulation of lymphocytes in the ME of late gestation sows which displays the phenotype of peripheral blood NK cells, but which exists in the pregnant uterus in far higher frequencies than NK cells in the blood [25]. The functional properties of porcine uNK cells specifically remain comparatively under investigated relative to human and murine uNK cells.

Porcine reproductive and respiratory syndrome virus (PRRSV) is a virus of considerable economic impact within the worldwide swine production industry causing reproductive failure in sows and retarded growth in piglets [26, 27]. Infection of a pregnant sow can lead to intrauterine infections when infected macrophages, the target cells of PRRSV, migrate to the uterus [28]. The pathogenicity of an intrauterine infection on the conceptus is dependent on the phase of gestation at the point of infection [29, 30]. Detection of viral infection in the conceptus tissue is rare in infections occurring in early to mid-gestation [31–33]. However, experimental infections during late gestation have demonstrated the ability of the virus to successfully infect the fetuses starting in the last third of gestation. The result of such late-stage gestation infections is often fetal death, stillbirths, or piglet death within 14 days of birth [31, 33]. Studies at the site of the maternal–fetal interface have found multifocal sites of increased apoptosis positively correlated with PRRSV viral load and associated with maternal–fetal interface micro-separations, though the exact mechanism of fetal death remains unclear as well as how the virus is able to cross the maternal–fetal barrier [34, 35]. Porcine uNK cells are known to be capable of killing trophoblast cells [36]. As it has been demonstrated that CD8α^+^ cells exist in significant concentrations near the uterine epithelium in PRRSV-positive samples, it has been suggested that uNK cells activated by a PRRSV infection may cause damage to trophoblast cells [37]. As it has been demonstrated that CD8α⁺CD2^+^ cells—likely corresponding to uterine NK cells—exist in increased numbers in the endometrium of PRRSV-positive sows [11], it possible that uNK cells activated by PRRSV infection may contribute to fetal compromise. This is supported by evidence showing that PRRSV infection induces apoptosis at the maternal–fetal interface and alters immune gene expression associated with inflammation and cell death [38].

In this study, we investigated the functional properties and subpopulation frequencies of porcine uNK cells isolated from the ME or FP of gilts in late-gestation which had been either experimentally infected with PRRSV or sham-infected on gestation day (gd) 85. Using flow cytometry, we defined three uNK cell subsets according to their NKp46-expression for the purposes of assessing relative subpopulation frequencies and evaluate variations according to the infection state of the sow and fetus. We used the degranulation marker CD107a and the cytolytic protein perforin to assess the cytolytic capacity of the uNK cells, as well as capacity to express IFN-γ and the proliferation marker Ki-67 to assess functional capacities of the uNK cells according to the infection state of the sow and fetus.

## Materials and methods

### Animal selection and experimental infection

Ten Landrace × Large White gilts recently delivered to an Austrian commercial piglet-producing farm were selected for this experiment. The farm is unsuspicious for PRRSV as confirmed through routine serological monitoring, and the gilts were confirmed PRRSV negative upon delivery to the farm by both a PRRSV-1 ORF1 RT-qPCR and IDEXX PRRS X3^®^ enzyme-linked immunosorbent assay (IDEXX PRRS X3^®^ Ab Test, IDEXX Europe B.V., Hoofddorp, the Netherlands). In accordance with the regular vaccination schedule of the source farm, all gilts were vaccinated against porcine parvovirus in combination with erysipelas (Parvoruvac^®^, Ceva Santé Animale, France) and against influenza virus (Respiporc FLU3^®^, Ceva Santé Animale) twice before breeding (4 weeks and 4 days prior), as well as against porcine circovirus type 2 prior to breeding (Ingelvac CircoFLEX^®^, Boehringer Ingelheim Vetmedica GmbH, Germany). The gilts were synchronized via hormonal application (Altrenogest, PMSG, and hCG). They were subsequently artificially inseminated (gd 0) with a follow-up pregnancy scan via ultrasound on gd 24. On gd 78 the gilts were transferred to the University of Veterinary Medicine, Vienna and were divided equally into two groups of five animals each. One group to be experimentally challenged with PRRSV and the other a control non-challenged group. The challenge group was housed in a biosafety level 2 (BSL-2) isolation unit, with the control group housed in the conventional housing outside of the BSL-2 unit. On gd 85 the challenge group was intranasally infected with the PRRSV-1 subtype 1 strain AUT15-33 (Gen-Bank accession number MT000052.1) at a concentration of 1 × 10^5^ TCID_50_ / mL with 2.5 mL administered per nostril using a mucosal atomization device. The isolate was propagated on porcine alveolar macrophages (PAMs) at the Institute of Virology of the University of Veterinary Medicine, Vienna. The control group received a cell culture supernatant from PAMs intranasally. The gilts and their litters were euthanized between gd 105–110, which corresponds to 19–23 days post-infection (dpi) whereby they were initially anesthetized by intravenous injection of Ketamine (Narketan^®^ 100 mg/mL, Vetoquinol Österreich GmbH, Vienna Austria, 10 mg/kg body weight) and Azaperone (Stresnil^®^ 40 mg/mL, Elanco GmbH, Cuxhaven, Germany, 1.5 mg/kg body weight) and subsequently euthanized via intracardial injection of T61^®^ (Intervet GesmbH, Vienna, Austria, 1 mL/kg body weight). Necropsies were performed, and tissue samples of the placental and endometrial tissues were collected. The experiment was approved by the institutional ethics and animal welfare committee (Vetmeduni Vienna) and the Austrian national authority according to §§26ff. of Animal Experiments Act, Tierversuchsgesetz 2012-TVG 2012 (accession number: 2021-0.117.108).

### Tissue sample collection and cell isolation

Samples of heparinized maternal blood (mBld) were collected from each gilt at four timepoints relative to the experimental challenge. The first collection was on the day of the experimental challenge, prior to infection (referred to as 0 dpi), followed by 7 dpi and 14 dpi and finally on the day of necropsy (19–23 dpi). At 0 dpi and 14 dpi, 70 mL of mBld was collected per gilt. On the day of necropsy, 200 mL of mBld was collected per gilt. Following each collection, the blood samples were used for the isolation of peripheral blood mononuclear cells (PBMCs) as previously described [25]. Following euthanasia, the uterus of the gilt was removed via an abdominal incision. The length of the anti-mesometrial side of the uterus was cut open to expose the fetuses. Three large non-necrotic fetuses were randomly selected from each gilt and the ME and FP for each fetus were extracted followed by digestion of tissue and isolation of lymphocytes as previously described [25]. Cells were counted on a Sysmex XP300 (Sysmex Austria GmbH, Vienna, Austria) and either used immediately (ex vivo phenotyping, IFN-γ assay, ME and FP samples of degranulation assay), or stored at −150 °C for future use (mBld PBMC samples for degranulation assay).

### NK cell degranulation assay

Isolated lymphocytes from the mBld PBMC, ME, and FP samples were diluted in a culture medium consisting of RPMI-1640, 10% (v/v) heat-inactivated fetal bovine serum (FBS, Sigma-Aldrich, Schnelldorf, Germany), 100 IU/mL penicillin (PAN-Biotech, Aidenbach, Germany), and 0.1 mg/mL Penicillin–Streptomycin (PAN-Biotech), and then pipetted into 96-well microtiter plates (Greiner Bio-One, Frickenhausen, Germany) at a concentration of 2 × 10^5^ cells in 150 µL culture medium per well. Each experimental sample described in the following was set up in three parallel wells for the incubation and stimulation period of the assay.

The cells were placed in an incubator at 37 °C and 5% CO_2_ for 14 h, after which a cytokine mixture of recombinant porcine (rp) IL-2 and rpIL-15 (R&D Systems, Minneapolis, MN, USA and Kingfisher Biotech, Saint Paul, MN, respectively) in 40 µL culture medium was added as a pre-activation step prior to exposure to target cells [39, 40]. Each cytokine was diluted to achieve a final concentration of 25 ng/mL.

After 24 h incubation, a mixture of anti-CD107a monoclonal antibodies (Table 1), 2 µg/mL Monensin, and 1 µg/mL Brefeldin A (both BD Biosciences, San Jose, CA, USA) were added to each well followed immediately by K562 (human erythroleukemia cell line) tumor cells serving as target cells for a final volume of 230 µL per sample [41]. K562 cells were continuously cultured in T75 flasks in RPMI− 1640, 10% (v/v) heat-inactivated FBS (Sigma-Aldrich), 100 IU/mL penicillin (PAN-Biotech), and 0.1 mg/mL Penicillin–Streptomycin (PAN-Biotech) during the experimental setup. The K562 target cells were added in an effector to target (E:T) ratio of 10:1, which was based on previous experiments to find the optimal ratio to induce degranulation across all three tissue types (data not shown). For each sample with target cells added, there was a paired control sample which was given an equal volume of culture medium. The cells were returned to the incubator for a final 4-h incubation, at the end of which the cells were centrifuged and the cell pellets of the three wells of each sample were re-suspended in culture medium and pooled into a single sample for flow cytometry staining.

**Table 1:** **Antibodies and streptavidin-conjugates used for FCM analysis**

Primary antibody	Clone	Isotype	Source	Labeling	Fluorophore
Degranulation assay					
CD107a	4E9/11	IgG1	BioRad	Direct	A647
CD8α	11/295/33	IgG2a	in-house	Indirect^A^	BV510
NKp46	VIV-KM1	IgG1	in-house	Indirect^B^^,^^C^	BV421
CD3	BB23-8E6	IgG2b	Southern Biotech	Indirect^D^	PE-Cy7
CD16	G7	IgG1	BioRad	Direct	PE
CD45	K252.1E4	IgG1	BioRad	Direct	FITC
IFN-γ assay					
CD8α	11/295/33	IgG2a	in-house	Indirect^A^	BV510
NKp46	VIV-KM1	IgG1	in-house	Indirect^B^^,^^C^	BV421
CD3	BB23-8E6	IgG2b	Southern Biotech	Indirect^D^	PE-Cy7
CD16	G7	IgG1	BioRad	Direct	FITC
CD45	K252.1E4	IgG1	BioRad	Direct	A647
IFN-γ	P2G10	IgG1	BD Bioscience	Direct	PE
Phenotyping					
CD8α	11/295/33	IgG2a	in-house	Indirect^B^^,^^E^	BV605
NKp46	VIV-KM1	IgG1	in-house	Direct	APC
CD3	BB23-8E6	IgG2b	Southern Biotech	Indirect^F^	PE
CD16	G7	IgG1	BioRad	Direct	FITC
Ki-67	B56	IgG1	BD Bioscience	Direct	BV421
Perforin	δG9	IgG2b	eBioscience	Direct	PerCP-eF710

^A^ Rat-anti-mouse anti-IgG2a BV510, BD Biosciences

^B^ Biotinylation bEZ-Link™ Sulfo-NHS-LC-Biotin, Thermo Fisher Scientific

^C^ Streptavidin BV421, BioLegend

^D^ Polyclonal goat anti-IgG2b PE-Cy7, Southern Biotech

^E^ Streptavidin BV605, BioLegend

^F^ Polyclonal goat anti-IgG2b PE, Southern Biotech

The cytokine stimulation we used during the incubation caused a portion of the NK cells to spontaneously degranulate even in the absence of K562 target cells. To control for this and to standardize the results, we subtracted the values from the control samples without target cells from their paired samples with target cells added. This allowed us to view the values of all samples as the degree of degranulation inducibility of the NK cells when exposed to target cells. We could then examine any difference in this inducibility to degranulate in the context of a PRRSV infection. Due to the applied cytokine stimulation, a clear separation of NKp46^+^ and NKp46^high^ subsets was not possible. As a result, we opted to analyze NKp46^−^ and NKp46^+/high^ subpopulations in our next analysis.

### IFN-γ assay

The freshly isolated lymphocytes from the mBld PBMC, ME, and FP samples were diluted with culture medium and pipetted into 96-well microtiter plates at a concentration of 2 × 10^5^ cells per well in 150 µL culture medium. Only freshly isolated mBld PBMC samples from the day of necropsy were used for this assay.

The cells were placed in an incubator at 37 °C and 5% CO_2_ for 14 h. Porcine NK cells do not spontaneously express IFN-γ without the addition of activating cytokines [39]. We utilized 50 µL stimulatory cytokine mixture of 25 ng/mL rpIL-2, 25 ng/mL rpIL-12, and 5 ng/mL rpIL-18 (R&D Systems) diluted in culture medium for a final well volume of 200 µL which we added to one of the two samples for each tissue type and location. The second sample received an equivalent volume of culture medium and served as an unstimulated control sample.

The cells were then incubated for 24 h, after which 1 µg/mL Brefeldin A was added to each well to inhibit the release of IFN-γ from the cells. The cells were returned to the incubator for a final 4- h incubation prior to FCM staining and FCM analysis described below. In our FCM analysis, we compared the frequencies of IFN-γ producing cells, as well as the expression levels of IFN-γ in the NK cells from PRRSV-infected gilts to those of the control gilts to assess if there is an observable difference after infection. Due to the applied cytokine stimulation, for this experiment we analyzed NKp46^+/high^ subpopulations together.

### Flow cytometry (FCM) staining

The ex vivo phenotyping samples of the ME and FP tissue samples were used immediately after isolation, samples for the IFN-γ and CD107a degranulation assay after culture, as described above. Control samples were prepared in the form of unstained samples, perforin fluorescence minus one (FMO) samples for the phenotyping assay, and IFN-γ FMO as well as unstimulated medium control samples for the IFN-γ samples.

Table 1 provides a detailed list of the antibodies used in each staining protocol. The antibodies used in the staining of the samples from the degranulation and IFN-γ assays were diluted in PBS + 3% (v/v) FBS, and those of the phenotyping were diluted in PBS + 10% (v/v) porcine plasma (in-house preparation) for a final volume of 20 µL per well. Following each addition of antibodies, samples were incubated at 4 °C for 20 min, after which they were washed twice with 200 µL of buffer. Surface markers were stained with either non-conjugated, biotinylated, or conjugated primary monoclonal antibodies (mAbs) followed by isotype-specific secondary antibodies or streptavidin conjugates as required. Fixable Viability Dye eFluor 780 (Thermo Fisher Scientific, Waltham, MA, USA) was applied to the degranulation and IFN-γ samples, and fixable Viability Dye eFluor 506 (Thermo Fisher Scientific) was applied to the phenotyping samples according to manufacturer’s protocols. When unconjugated and conjugated mAbs with the same isotype were used in the same sample, whole mouse IgG molecules (2 µg/sample, ChromPure, Jackson ImmunoResearch, West Grove, PA, USA) were added to block free binding sites after application of the indirect staining, before applying directly-labelled mAbs. Following these steps, samples from all three stainings were fixed and permeabilized. For the degranulation and IFN-γ assay samples, this was done with BD Cytofix/Cytoperm Kit (BD Biosciences), and for the phenotyping samples with eBioscience Fixation/Permeabilization Kit (Thermo Fisher Scientific) according to manufacturer instructions. The IFN-γ and phenotyping stainings then had final intracellular staining steps with either anti-IFN-γ, or for the phenotyping samples anti-Ki-67 and anti-perforin mAbs.

### FCM analysis

The samples from all three staining panels were analyzed on a FACSCanto II (BD Biosciences) or CytoFLEX LX (Beckman Coulter GmbH, Krefeld, Germany) flow cytometer. Data were analyzed with FACSDIVA software (Version 8.0, BD Bioscience), and FlowJo software (Version 10.8.1, BD Biosciences). A consecutive gating strategy was used to identify cells consistent with an NK cell phenotype (Additional file 1). Lymphocytes were identified through their light scatter properties using forward and side scatter area (FSC-A, SSC-A), followed by a 2-step doublet discrimination using FSC-height (FSC-H) against FSC-A and SSC-H against SSC-A. Viable cells were selected using a viability dye (eFluor780^®^ for the IFN-γ and degranulation samples, eFluor506^®^ for the phenotyping samples).

### Virology analysis

A quantitative analysis of mBld, placental tissue, and maternal endometrium samples were analyzed using an in-house RT-qPCR protocol targeting the ORF1 gene region of the viral genome as previously described [25]. The detection threshold was 10^4^ genome equivalents per mL or gram tissue.

### Statistical analysis

All hypothesis testing was performed in *R v4.2.1* [42]. Data was prepared with functions in packages *dplyr version 1.1.4* and *tidyr version 1.3.0* [43, 44]. We declare significance at 10% False Discovery Rate (FDR) multiple testing correction [45].

We fitted univariate linear mixed models applying function *lmer* in R package *lme4 v1.1–35.1* allowing for 100 000 iterations to reach convergence via maximum likelihood estimation to get best estimates for the fixed effects parts of the models by setting option *REML* to false [46].

To account for the covariance structure in our data caused by multiple measurements taken from the same animals we fitted “gilt” as random intercept with 10 factor levels, five control gilts and five PRRSV-infected gilts, respectively. To reduced residual variance a random intercept of day of experiment / sample collection was added to all the models as well.

Homoscedasticity of residuals, and normal distribution of residuals and fitted random intercepts and slopes, where applicable, were verified with custom R scripts.

We then calculated estimated marginal means and pairwise differences for contrasts of interest using function *emmeans* in package *emmeans v1.8.*9 with option *pairwise*. Default multiple testing correction was turned off with option *adjust* = *“none”*. Multiple testing correction was then applied for contrasts of interest across all response variables within a group of phenotypes [47].

We visualized results of our hypothesis testing models as bar plots produced with packages *RColorBrewer v1.1–3*, *ggplot2 v3.4.4* and *ggpubr v0.6.0* [48–50]. Estimated marginal means are shown as the height of the plot and whiskers depict their upper and lower 95% confidence intervals. P-value brackets are drawn for contrasts that are significant at 10% FDR. Figures were exported as scalable vector graphics using package *svglite v2.1.3* [51].

Frequencies of NKp46-defined NK subsets, NKp46^neg^, NKp46^pos^, and NKp46^high^, are compositional data that measure relative rather than absolute information whose proportions or percentages sum up to 1 or 100%. To make compositional data amendable for univariate modelling we applied a centered log ratio (clr) transformation to get rid of the constant-sum constraint using function *clr* in package *compositions v2.*0–6 [52].

All other phenotypes were log_10_ transformed before hypothesis testing and if a phenotype included measures of zero we added a constant of one to all observations before transformation.

#### Comparisons between tissues

Our models included main effects of fixed categorical effect “infection status” with two factor levels -control and PRRSV-infected, and a fixed categorical effect of “tissue” with three factor levels mBld PBMC, ME and FP, respectively. Key in our hypothesis testing models is a fixed effects interaction between these two main effects.

As levels of our random effect “gilt” vary across the levels of our fixed categorical effect “tissue”, we fitted dummy coded and centered random slopes for “tissue”, to ensure we keep false-positive declarations at our predefined significance cutoff alpha as recommended by Barr et al. [53].

#### Time series data measured only in mBld PBMC

This model included main effects of fixed categorical effect “infection status” with two factor levels -control and PRRSV-infected, and a fixed categorical effect of “day” with four factor levels—day 0 prior to infection, day 7, 14 after infection and day of necropsy (19–23 dpi), respectively. Key in these hypothesis testing models is a fixed effects interaction between these two main effects.

#### CD107a expression

Hypothesis testing models for CD107a expression additionally included the measures with no K562 target cells as covariate, in the models comparing different tissues the covariate was scaled and centered.

#### IFN-γ expression

Hypothesis testing models for IFN-γ expression additionally included IFN-γ measures in the medium control samples as scaled and centered covariate.

## Results

### PRRSV infection influences NK cell proliferation marker expression but not NK cell population frequencies

**Figure 1:**
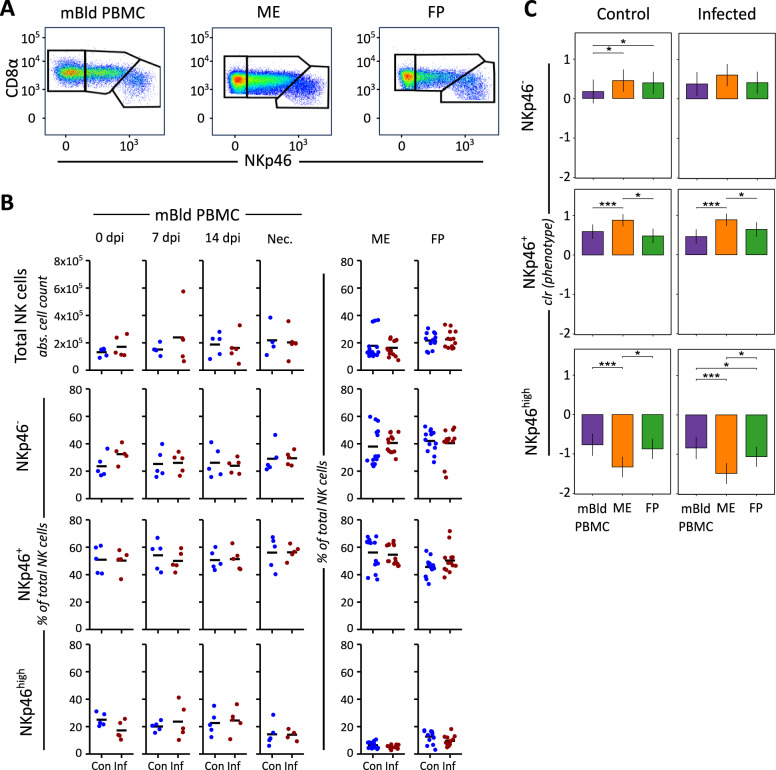
**Porcine NK cell population sizes within total lymphocytes and frequencies of subpopulations based on NKp46 expression.** **A** Representative flow cytometry dot plots showing NK cell subpopulation gating based on NKp46 expression. **B** Population sizes of total NK cells and NKp46-defined cell subpopulations of samples from control (Con) and PRRSV- infected (Inf) gilts. Population measurements of NK cells from three different tissue origins are shown: maternal blood NK cells (mBld NK) selected from peripheral blood mononuclear cells (PBMC) isolated from maternal blood samples, and putative uterine NK cells (uNK) selected from lymphocytes isolated from maternal endometrium (ME) and fetal placenta (FP) samples taken from the day of necropsy. The total NK cells in the mBld PBMC samples are given in calculated absolute cell counts. For the total NK cells of ME and FP samples from day of necropsy, this is given as frequency of NK cells within total measured lymphocytes. The NKp46-defined subpopulations of all three tissues are given in frequency of total measured NK cells. The median value of each subgroup is depicted with a black bar. **C** Univariate compositional data analysis was performed for the three NKp46-defined subpopulations. The y-axis depicts the estimated marginal means (emmeans) of the centered log ratios (clr) transformed NKp46^−^, NKp46^+^, and NKp46^high^ NK cell subpopulations for the three tissues and two experimental conditions. Only significant *P*-values are shown (*P* < 0.1), corrected for multiple testing using a false discovery rate approach, across all pairwise comparisons of contrasts for all (18) compositional cell subpopulations and all three tissues. The whiskers depict the 95% confidence intervals of the emmeans. *P < 0.1,**P < 0.01,***P < 0.001.

To investigate qualitative changes in NK cell populations in the context of a PRRSV infection, we analyzed mBld PBMC samples from both control and PRRSV-infected animals across four time points (dpi 0, 7, 14, and necropsy) as well as from the ME and FP at the point of necropsy. Total NK cells were further subdivided into three subsets based on their NKp46 and CD8α expression: NKp46⁻, NKp46⁺, and NKp46^high^ (Figure 1A). In the mBld PBMC longitudinal study, we compared the frequencies of total NK cells and each NKp46-defined subset between control and PRRSV-infected animals at each time point. Median frequencies did not differ significantly between the two groups over time, indicating that PRRSV infection did not result in measurable changes in the relative abundance of these NK cell populations in peripheral blood during the period examined (Figure 1B). At the point of necropsy, the ME and FP samples also showed no significant variation within tissue types between control and PRRSV-infected groups (Additional file 2). Following clr transformation of the NKp46-defined subpopulation data, a comparison between the three tissues showed that ME uNK cells had the highest population sizes in the NKp46^+^ subpopulation and the lowest in the NKp46^high^ subpopulation in both the control and PRRSV-infected samples relative to the NK cell populations in the other tissues examined (Figure 1C). The mBld NK cell population size was significantly lower in the NKp46^−^ subpopulation relative to the other two tissues in the control samples, however, there was no significant difference between the tissues in this subpopulation in the samples of PRRSV-infected gilts. The mBld NK cell and FP relative population sizes were comparable in the NKp46^+^ subpopulations of both control and PRRSV-infected samples, as well as in the NKp46^high^ subpopulation of the control samples. In the NKp46^high^ subpopulations of the PRRSV-infected samples, a decrease in the frequency of the FP samples resulted in a significant difference between the FP and mBld samples.

**Figure 2:**
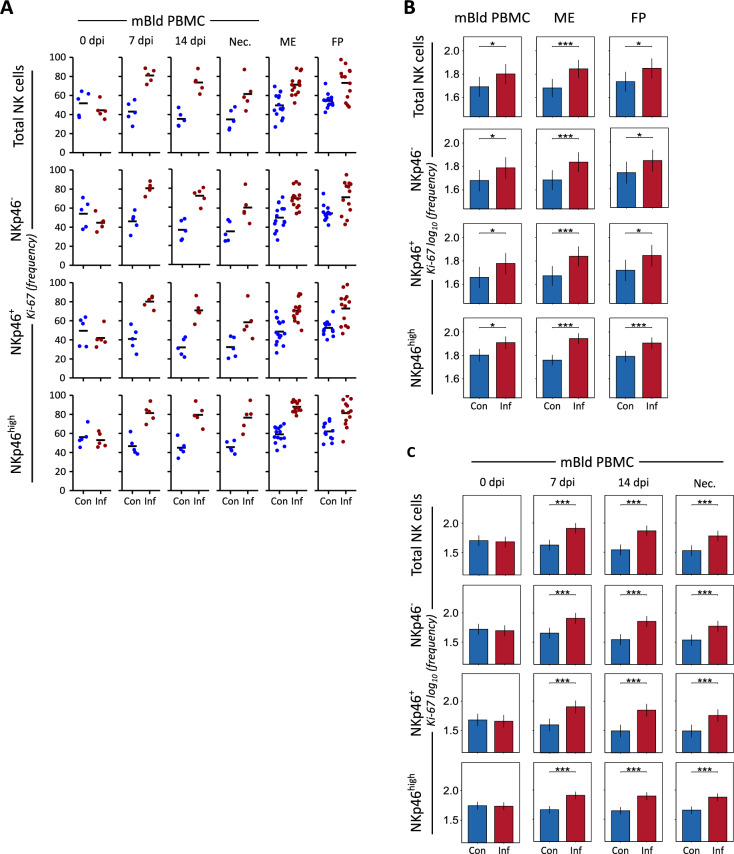
**Expression of the proliferation marker Ki-67 within porcine NK cells.** **A** Expression of Ki-67 within total NK cells and NKp46-defined subpopulations. NK cells are mBld NK cells from four timepoints following experimental or sham infection, and putative uNK cells from day-of-necropsy ME and FP samples. The median value of each subgroup is depicted with a black bar. **B** A linear mixed effects model considering infection status, tissue, and the interaction between both was applied. A random intercept (gilt) was fitted and estimated marginal means (emmeans) were calculated using measured population Ki-67 expression. The y-axis depicts results obtained from the three NKp46-defined subpopulations as well as total NK cells in a log_10_ transformed scale. Only significant *P*-values are shown (*P* < 0.1). The whiskers depict the 95% confidence intervals of the emmeans. **C** A linear mixed effects model considering infection status, days elapsed since experimental or sham infection, and the interaction between both was applied. A random intercept (gilt) was fitted and estimated marginal means (emmeans) were calculated using measured population Ki-67 expression. The y-axis depicts results obtained from the three NKp46-defined subpopulations as well as total NK cells in a log_10_ transformed scale. Only significant *p*-values are shown (*P* < 0.1). The whiskers depict the 95% confidence intervals of the emmeans. *P < 0.1,**P < 0.01,***P < 0.001.

To further evaluate proliferation changes in the NK populations in the gilt blood and reproductive tissue, we examined NK cell expression of the proliferation marker Ki-67 [54]. In FCM stainings conducted on unstimulated isolated lymphocytes, we identified the cells expressing the phenotype perforin^+^CD3^+^CD8α^+^CD16^+^ and evaluated the frequency of Ki-67 positive NK cells in the control and PRRSV-infected groups within total NK cells, as well as subdivided into three groups based on the NK cell activating receptor NKp46 (Figure 2A and Additional file 3). At 7 dpi, the proportion of Ki-67 positive NK cells in the PRRSV-infected mBld samples showed a clear increase compared to the control samples with a median value of over 80% of NK cells expressing Ki-67 in the PRRSV-infected samples. After this peak, the proportion of Ki-67 positive NK cells in the PRRSV-infected mBld samples gradually declined across subsequent timepoints in the total NK cell population, as well as within both the NKp46^−^ and NKp46^+^ subpopulations. Nevertheless, Ki-67 positive NK cells continued to represent a larger share of the total NK cell population than they did at 0 dpi. The NKp46^high^ subpopulation of the PRRSV-infected group, in contrast, did not show a decrease in median frequency levels from 7 dpi through to the point of necropsy. The median fluorescence intensity (MFI) of Ki-67 within the PRRSV-infected samples showed a notable increase relative to the control samples at 7 dpi, however, this difference in MFI was not evident in any group except for NKp46^high^ by the next measurement at 14 dpi (Additional file 4A).

NK cells in the control samples from both the ME and FP tissue samples displayed comparable proportion of Ki-67 positive cells**,** whereas NK cells from the PRRSV-infected group samples showed a higher median proportion of Ki-67 positive NK cells (Figure 2A). However, considerable individual variation within each examined NK cell population—across both tissue types and samples from both control and PRRSV-infected groups—limited the statistical significance of the increased frequency of Ki-67-expressing NK cells observed in the PRRSV-infected group. This variation was most notable in the FP samples from the PRRSV-infected group. In contrast, the Ki-67 MFI of the ME and FP samples from the control group showed much less individual variation within total NK cells and each subpopulation examined. This reduction in individual variation was also observed in the ME samples from the PRRSV-infected group for total NK cells as well as both NKp46^−^ and NKp46^+^ NK cell subsets. However, the Ki-67 MFI within the NKp46^high^ subpopulation of these samples showed a much wider spread of measured values (Additional file 4B). The FP samples from the PRRSV-infected group of all examined NK cell populations exhibited considerably greater variation in Ki-67 MFI than those of the control samples. However, this increase in variation was only accompanied by a small increase in the median values of each NK cell population when compared to the median values from the control samples.

We summarized the proportion of Ki-67 positive NK cells for all day-of-necropsy samples within each Nkp46 subset and tissue and compared the PRRSV-infected and control group samples and found a significant increase in Ki-67 positive NK cells in the PRRSV-infected group samples for all (Figure 2B). We similarly summarized the data from the mBld PBMC longitudinal studyand found a significant increase in Ki-67 positive NK cells in all PRRSV-infected group samples from 7 dpi through necropsy in the case of total NK cells and all NKp46-defined subgroups (Figure 2C).

### PRRSV infection causes greatest increases in perforin MFI in NKp46^high^ NK cells

Previous research has described perforin expression to be a part of the porcine NK cell phenotype [21, 24, 55]. We previously confirmed that this universal porcine NK cell expression of perforin extends to uNK cells as well, indicating a capacity for cytolytic activity despite the supportive non-cytolytic role played by uNK cells in early pregnancy [25]. In this investigation, we focused on a quantitative measurement of perforin MFI in the control samples compared to the PRRSV-infected group samples (Additional file 5).

**Figure 3:**
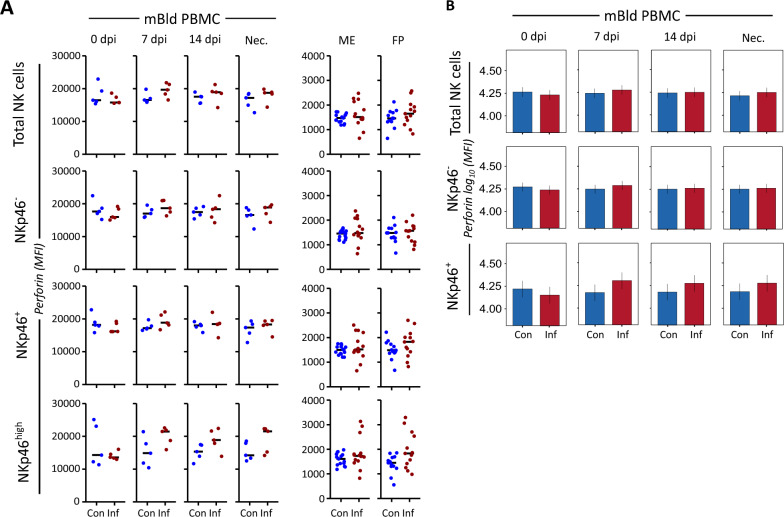
**Mean fluorescence intensity (MFI) of perforin within porcine NK cells of PRRSV-infected and control samples by dpi and NKp46 subpopulation.** **A** Measured perforin MFI within NK cells of individual samples. The rows show total NK cells as well as NKp46-defined subpopulations. The columns show mBld NK cells at four time points after experimental or sham infection including day of necropsy, and ME and FP samples from day of necropsy. The median value of each subgroup is depicted with a black bar. **B** A linear mixed effects model considering infection status, days elapsed since experimental or sham infection, and the interaction between both was applied. A random intercept (gilt) was fitted and estimated marginal means (emmeans) were calculated using measured population perforin MFI values. The y-axis depicts results obtained from the three NKp46-defined subpopulations as well as total NK cells in a log_10_ transformed scale. Only significant *P*-values are shown (*P* < 0.1). The whiskers depict the 95% confidence intervals of the emmeans. *P < 0.1,**P < 0.01,***P < 0.001.

In the mBld PBMC longitudinal study, we did not observe significant differences in the MFI of perforin across groups within total, NKp46^−^, or NKp46^+^ NK cells (Figure 3A). Within the NKp46^high^ NK cells, we observed an increase at 7 dpi onwards until necropsy in the median value recorded from the PRRSV-infected group samples compared to the value measured at 0 dpi from the same samples, although not significant. The median value of control samples remained constant throughout the study. However, the high variability within both groups prevented us from detecting significant differences between the PRRSV-infected and control samples (Figure 3B).

We did not observe a significant difference in the median perforin MFI levels between the PRRSV-infected and control groups of either ME or FP samples (Figure 3A and Additional file 6). However, within the ME and FP tissue samples we did observe a higher heterogeneity of perforin MFI levels in the PRRSV-infected group samples compared to the control samples, in particular within the PRRSV-infected group ME samples.

### uNK cells of the FP show least capacity to degranulate, regardless of infection status

The degranulation marker CD107a has previously been shown to be an effective measure of NK cell cytolytic activity [41, 56]. To evaluate the cytotoxic capacity of NK cells in the context of a PRRSV infection, we applied a cytokine stimulation (IL-2 + IL-15) to isolated lymphocytes for 24 h for pre-activation and then added K562 target cells in an E:T ratio of 10:1 and measured the expression of the degranulation marker CD107a in NK cells (Additional file 7).

**Figure 4:**
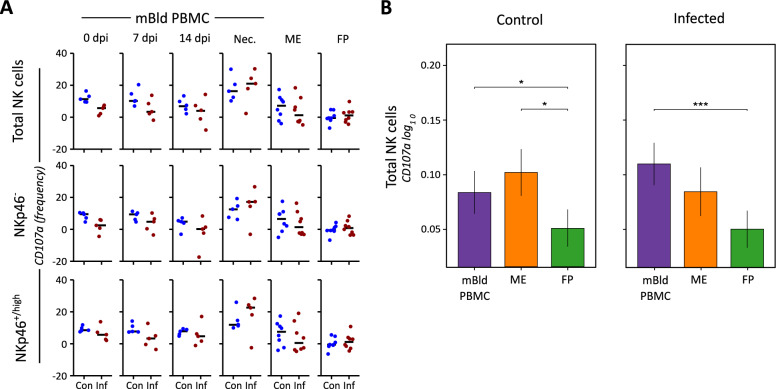
**Frequency of expression of CD107a in porcine NK cells.** **A** Measured CD107a expression frequency of the samples with no K562 target cell has been subtracted from the matched samples to which K562 target cells were added. The NK cell populations are presented as total NK cells, as well as NKp46^−^ and NKp46^+/high^ subpopulations. The columns show mBld NK cells at four different timepoints post experimental or sham infection, and putative uNK cells isolated from ME and FP samples collected on day of necropsy. **B** A linear mixed effects model considering infection status, tissue, and the interaction between both was applied. A random intercept (gilt) was fitted and estimated marginal means (emmeans) were calculated using the measured population. The y-axis depicts the estimated marginal means (emmeans) of the log_10_ transformed NKp46^−^ and NKp46^+/high^ NK cell subpopulations for the three tissues and two experimental conditions. Only significant *P*-values are shown (*P* < 0.1), corrected for multiple testing using a false discovery rate approach, across all pairwise comparisons of contrasts for both compositional cell subpopulations and all three tissues. The whiskers depict the 95% confidence intervals of the emmeans. *P < 0.1,**P < 0.01,***P < 0.001.

Figure 4A presents the data from the degranulation assay after adjusting for background CD107a expression by removing the values obtained from no-K562 samples from their corresponding K562-paired samples. Within the timeframe of 0 dpi to 14 dpi for mBld PBMC samples, a slight fluctuation in CD107a levels on NK cells was noted, however, there was no statistically significant change between any sample groups or timepoints. Control group samples showed a rise in CD107a expression from 14 dpi to necropsy, mirroring the increase observed in PRRSV-infected gilts between these same timepoints. Nonetheless, this increase at necropsy did not mark a statistically significant difference between the groups. Distinct from the pattern observed in mBld PBMCs, ME samples from PRRSV-infected gilts exhibited a reduced median expression of CD107a compared to those from the control group. For FP samples, both the infected and control groups demonstrated similar median expressions, with this consistency also featuring less variability than seen in ME and mBld PBMC samples.

A comparison of degranulation on the day of necropsy by tissue found the FP samples to be significantly lower than either ME uNK cells or mBld NK cells in the control samples (Figure 4B). The difference between ME and FP samples within the PRRSV-infected group was not found to be significant due to a reduction in the ME samples relative to the control ME samples, but the trend remained. The FP samples showed almost no change between control and PRRSV-infected samples.

### IFN-γ MFI of ME uNK cells was reduced in presence of PRRSV infection

**Figure 5:**
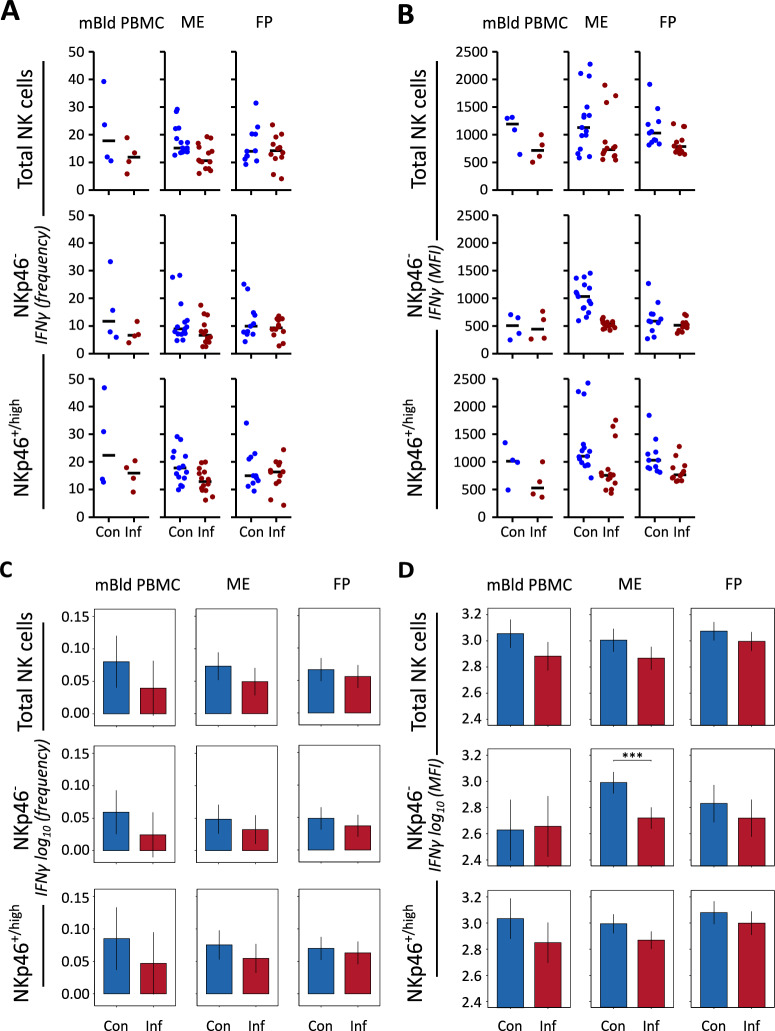
**Expression of IFN-γ in NK cells isolated from maternal blood, ME, and FP tissue samples collected on day of necropsy.** **A** Percentage of total NK cells and NKp46^−^ or NKp46^+/high^ NK cell subpopulations expressing IFN-γ in individual samples. The median value of each subgroup is depicted with a black bar. **B** MFI of IFN-γ in total NK cells and NKp46^−^ or NKp46^+/high^ NK cell subpopulations in individual samples. The median value of each subgroup is depicted with a black bar. **C** A linear mixed effects model considering infection status, tissue, and the interaction between both was applied. A random intercept (gilt) was fitted and estimated marginal means (emmeans) were calculated using the measured population IFN-γ expression frequencies. The y-axis depicts results obtained from the two NKp46-defined subpopulations as well as total NK cells in a log_10_ transformed scale. Only significant *P*-values are shown (*P* < 0.1). The whiskers depict the 95% confidence intervals of the emmeans. **D** A linear mixed effects model considering infection status, tissue, and the interaction between both was applied. A random intercept (gilt) was fitted and estimated marginal means (emmeans) were calculated using the measured population IFN-γ MFI values. The y-axis depicts results obtained from the two NKp46-defined subpopulations as well as total NK cells in a log_10_ transformed scale. Only significant *P*-values are shown (*P* < 0.1). The whiskers depict the 95% confidence intervals of the emmeans. *P < 0.1,**P < 0.01,***P < 0.001.

To further clarify the immune actions of porcine NK cells in response to PRRSV infection, we examined the expression of the pro-inflammatory cytokine IFN-γ. The NK cell IFN-γ expression within the control samples was characterized by considerable individual variation across all three tissue types (Figure 5A). Observed variation was greatest within the mBld NK cells. This variation between tissue-matched samples stands in contrast to the frequencies measured in the samples from PRRSV-infected gilts. In these samples, we observed both a reduction in variation between samples, as well as a reduction in the median level of IFN-γ^+^ NK cells in mBld PBMC and ME samples. The results of the FP samples showed comparable levels of expression and individual variation between the two groups. Although a clear trend (reduction in frequencies) was observed for mBld and ME NK cells, differences were not significant (Figure 5C). The MFI data for these measurements showed even less variation within ME samples from PRRSV-infected gilts as well as a greater contrast in the median measured value compared to the control samples (Figure 5B). When looking at the MFI of IFN-γ expression we can see a trend towards reduced values of PRRSV-infected samples compared to controls in most samples, however, only the NKp46^−^ ME uNK cells showed a significant difference between treatment groups (Figure 5D).

## Discussion

The contributions of uNK cells during early pregnancy have been extensively studied in humans and mice [57–61], but their role in later gestation remains less well understood. In murine pregnancy, a decline in both absolute numbers and relative proportion of uNK cells has been observed after placental formation, despite their early dominance in the decidua [10, 62–64]. Our previous work demonstrated that in late-stage gestation of healthy sows, uNK cells remain prominent in ME and FP tissues, comprising a significantly greater proportion of lymphocytes than in mBld [25]. In the current study, we did not observe consistent differences in total NK cell counts between PRRSV-infected and control animals, due to high individual variation in the samples (Figure 1B). However, analysis of the proliferation marker Ki-67 revealed a significant increase in the proportion of Ki-67 positive cells among mBld NK cells in the PRRSV-infected group at 7 dpi (Figure 2A), which remained elevated through 14 dpi and necropsy. Similarly, uNK cells from ME and FP tissues showed increased proportions of Ki-67 positive NK cells at necropsy, with the largest increases in the NKp46^high^ subset. Notably, high NKp46 expression has previously been linked to co-expression of CD27, a marker of less mature NK cells, suggesting that these cells may represent a more developmentally immature population [39, 65]. This corresponds with previous reports of peak PRRSV viremia occurring around 7–8 dpi [66, 67], and with studies showing an initial decline in mBld NK cell numbers post-infection, followed by a rebound beginning around 6 dpi and continuing through 19 dpi [68]. These dynamics may explain the lack of significant group-level differences in NK cell counts at 7 dpi despite increases in Ki-67 positive NK cells. Notably, Ki-67 MFI was also elevated in several infected samples at 7 dpi, particularly in NKp46^high^ cells, but returned to baseline by 14 dpi. This transient elevation suggests a short-lived proliferative burst in response to initial viral exposure. The MFI patterns in FP samples further support a later-stage encounter with the virus, consistent with a progression of PRRSV dissemination from mBld to ME and ultimately to the FP tissue.

NKp46^high^ porcine NK cells are known to exhibit heightened responsiveness to stimulation, which may account for the pronounced upregulation of perforin observed in this subset [39]. In our study, NKp46^high^ cells from PRRSV-infected mBld samples showed increased perforin MFI compared to controls, consistent with prior reports of PRF_1_ gene upregulation in PRRSV-infected lung NK cells [69]. This increase emerged at 7 dpi and persisted through necropsy (Figure 3B). At necropsy, the NKp46^high^ mBld NK cells from the PRRSV-infected group exhibited perforin levels comparable to those of NKp46^high^ uNK cells in PRRSV-infected FP tissue (Additional file 6), suggesting a systemic cytolytic response associated with infection.

As not all fetuses from the PRRSV-infected group had detectable viral genetic material in their placental tissue, we subdivided the samples based on viral presence at necropsy. Two FP samples were virus-negative, likely reflecting the expected progression of PRRSV infection from the ME to the FP, suggesting these fetuses had not yet been exposed. These virus-negative samples showed similar perforin MFI levels. In contrast, FP samples with detectable virus tended to show equal or higher perforin MFI across all NKp46-defined subsets. While the small sample size prevents definitive conclusions, this pattern suggests that local viral exposure may enhance perforin expression in uNK cells.

To our knowledge, the capacity of porcine uNK cells to degranulate in uterine tissues has not previously been examined. In this study, we compared the degranulation capacity, as measured by surface expression of CD107a [41, 70–73] of NK cells isolated from mBld PBMC, ME, and FP tissues, and evaluated whether PRRSV infection affected this response.

We observed that uNK cells from FP samples displayed the lowest capacity to degranulate under the assay conditions, potentially reflecting the more isolated and immature fetal immune environment compared to maternal tissues (Figure 4B). As the assay included all mononuclear leukocytes isolated from the tissues, the reduced response may also indicate weaker paracrine activation signals within the FP microenvironment. Notably, PRRSV infection did not significantly alter this limited degranulation capacity, as CD107a expression was nearly identical between infected and control group FP samples across total uNK cells and both NKp46-defined subsets measured in the assay (Figure 4A).

In human uNK cells, previous research has reported lower degranulation capacity in first-trimester ME uNK cells compared to mBld NK cells, however, degranulation capacity of IL-15 stimulated ME uNK cells at term was found to be much higher and comparable to mBld NK cells [74]. In our study, although we did not find significant differences in CD107a expression between ME and mBld NK cells overall (Figure 4B), ME uNK cells from PRRSV-infected animals showed a non-significant trend toward reduced median CD107a expression compared to controls (Figure 4A). This aligns with earlier findings showing diminished NK cell cytotoxicity following exposure to PRRSV-infected macrophages [75]. We also noted a slight reduction in CD107a expression among mBld NK cells at 7 dpi in PRRSV-infected gilts, though baseline values for these animals were already slightly lower than controls at 0 dpi. This subtle difference is consistent with previously observed individual variability in CD107a expression among uninfected gilts exposed to K562 target cells (unpublished data).

IFN-γ plays a context-dependent role in uNK cell function. During placental development, it promotes spiral artery remodeling and pro-angiogenesis [64], in contrast to its aggressive anti-angiogenic and vessel-destructive role in pbNK cells [76–78]. At the maternal–fetal interface, IFN-γ is also involved in immune cell recruitment and differentiation [79]. In early pregnancy, a surge in IFN-γ expression peaks during peri- to post-implantation, with uNK cells identified as key contributors based on uNK-depletion studies and their temporal correlation with IFN-γ production [80].

We examined whether PRRSV infection alters IFN-γ expression in NK cells from mBld, ME, and FP tissues. Across all compartments, median IFN-γ expression was generally lower in the PRRSV-infected group than in controls, though high individual variability precluded statistical significance (Figure 5A). MFI values reflected a similar trend, with clearer differences between groups. Notably, IFN-γ MFI values in PRRSV-infected samples were more tightly clustered, while control samples showed broader variability. A significant reduction was observed only in ME NKp46⁻ uNK cells, likely due to greater variance in the control group. These findings align with previous reports of suppressed NK cell function in response to PRRSV [74]. Only one tissue subset across all measurements showed matching median MFI values between experimental and control groups (Figure 5D).

Our previous work demonstrated that a PRRSV infection can induce a change in lymphocyte phenotypes, including that of uNK cells. Here, show that infection also induces transient proliferative and functional changes in uNK cells within the ME and FP. Although overall NK cell counts remained unchanged, elevated Ki-67 expression indicates a short-lived proliferative response to infection. Functionally, our findings suggest a complex immunomodulatory effect: while markers of cytotoxic activity such as CD107a and IFN-γ expression were generally suppressed, perforin levels—particularly in NKp46^high^ subsets—were elevated. These findings support the idea that highly virulent PRRSV strains may drive both immune activation and immune dysregulation at the maternal–fetal interface, with implications for viral pathogenesis and fetal health.

## Supplementary Information

**Additional file 1 Gating strategy for identifying porcine NK cells.** **A** Gating strategy for maternal blood samples, **B** maternal endometrium, and **C** fetal placenta tissue samples. For each of the three staining panels used in this study, lymphocytes were selected according to their light scattering properties (SSC-A against FSC-A). This was followed by a two-step doublet discrimination (SSC-H against SSC-A, and FSC-H against FSC-A), and subsequently CD8α was gated first against an empty channel to eliminate autofluorescence in the sample, and then against a viability dye. NK cells were then specifically selected by gating on CD16^+^/CD3^-^ cells, followed by selecting all cells which displayed a CD8α^+^/NKp46^-^ or NKp46^+^ phenotype as well as those displaying a CD8α^dim^/NKp46^high^ phenotype. The expression threshold between Ki-67^+^ and Ki-67^-^ cells within the NK cells was often difficult to determine. We therefore set the gate for Ki-67^+^ according to a group of non-NK cells showing a CD16^-^/CD3^-^ phenotype which showed a clear +/− separation for this marker. The representative samples presented here were from freshly isolated, unstimulated phenotyping samples.

**Additional file 2 NK cell population within total lymphocyte population.** A linear mixed effects model considering infection status, tissue, and the interaction between both was applied. A random intercept (gilt) was fitted and estimated marginal means (emmeans) were calculated using measure population frequencies. The y-axis depicts the population of NK cells relative to total lymphocytes from each respective tissue group in a log_10_ transformed scale. Only significant *p*-values are shown (*P* 0.1). The whiskers depict the 95% conﬁdence intervals of the emmeans.

**Additional file** **3 Representative gating for Ki-67 expression within total NK cells and NKp46-defined NKsubpopulations.** The percentage of positive cells from the sample total is given above the line representing the zoneof positive expression.

**Additional file 4 MFI of Ki67 expression**. **A** MFI of Ki67 from maternal PBMCs from days 7 dpi and 14 dpi. The median value of each subgroup is depicted with a black bar. **B** MFI of Ki67 from putative uNK cells isolated on day of necropsy from ME and FP tissue samples. The median value of each subgroup is depicted with a black bar.

**Additional file 5 Dot plots of perforin expression frequency within NK cells**. Dot plots demonstrating the universal expression of perforin within the total NK cell population and NKp46-defined subpopulations of all three tissue types measured. Y-axis is forward scatter area (FSC-A).

**Additional file 6 MFI of perforin expressed in FP and maternal PBMCs isolated on day of necropsy, grouped into NKp46-defined subpopulations.** A linear mixed effects model considering infection status, tissue, and the interaction between both was applied. A random intercept (gilt) was fitted and estimated marginal means (emmeans) were calculated using measured population perforin MFI values. The y-axis depicts the population of the NKp46-defined NK cell subpopulation relative to total NK cells from each respective tissue group and infection status in a log_10_ transformed scale. The whiskers depict the 95% conﬁdence intervals of the emmeans.

**Additional file 7 Representative gating for CD107a expression within total NK cells and NKp46-defined NK subpopulations. **The grey shaded area represents the sample with no K562 target cells added, and the unshaded area with the thick outline represents the sample with the K562 target cells added in a target cell to effect cell ratio of 10 to 1. The value above the gating line is the value recorded from the sample with target cells, and the value below the line is the value recorded from the control sample without target cells.

**Additional file 8 Representative gating for IFNγ expression within total NK cells and NKp46-defined NK subpopulations**. The grey shaded area represents the unstimulated sample incubated in only cell culture medium, and the unshaded area with the dark outline represents the sample incubated in a cytokine stimulation mixture of IL-2, IL-15, and IL-18. The value above the line represents the value recorded from the stimulated sample, and the value below the line represents the value recorded from the unstimulated sample.

## Data Availability

The raw data supporting the conclusions of this article will be made available by the authors, without undue reservation.
