# Supplementary figures and images for: Investigating the functional capacity of porcine uterine natural killer cells during a porcine reproductive and respiratory syndrome virus infection of pregnant gilts
Source: Vet Res. 2025 Oct 7;56:189. doi: 10.1186/s13567-025-01623-8 (PMC12506264; doi:10.1186/s13567-025-01623-8)

**A**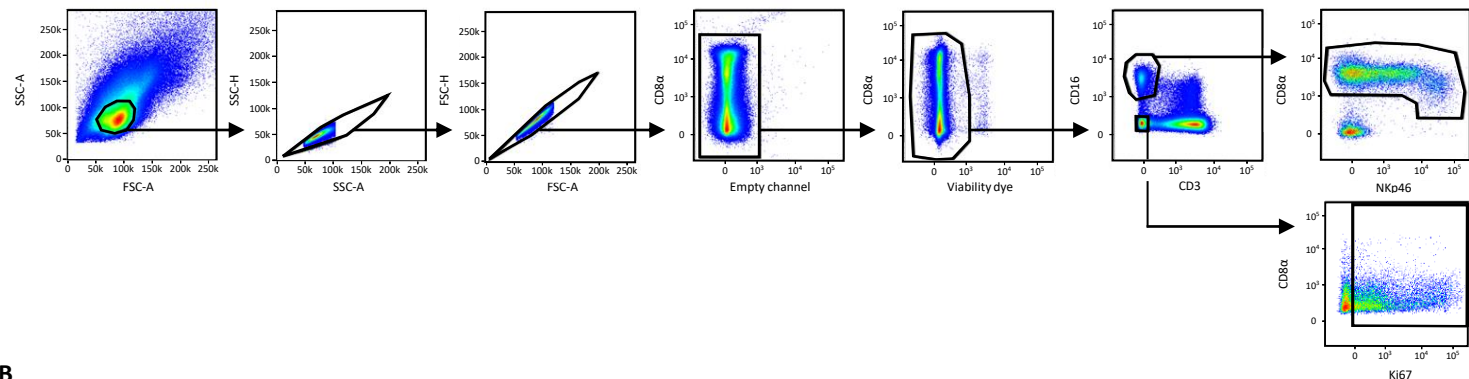**B**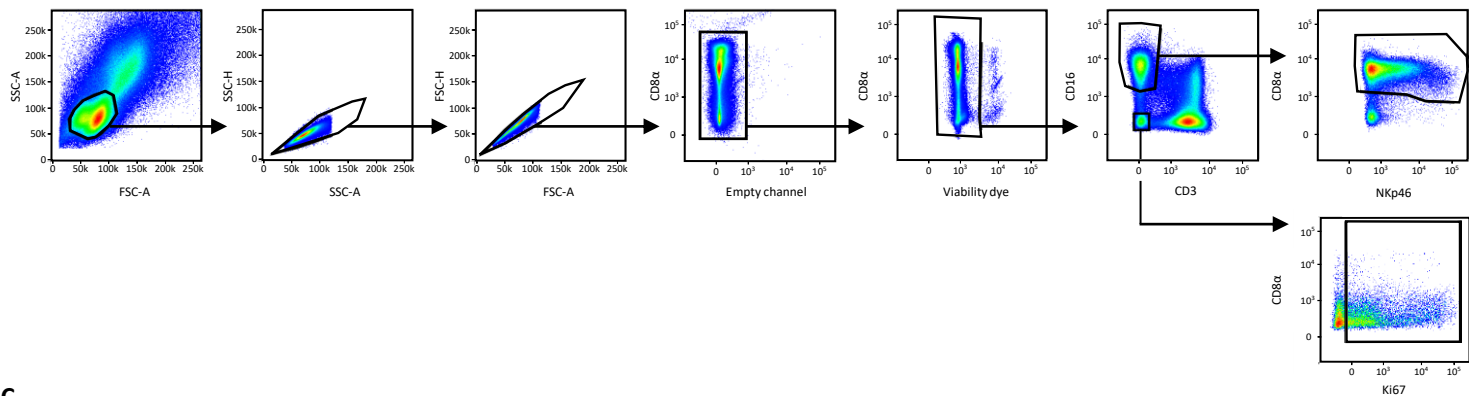**C**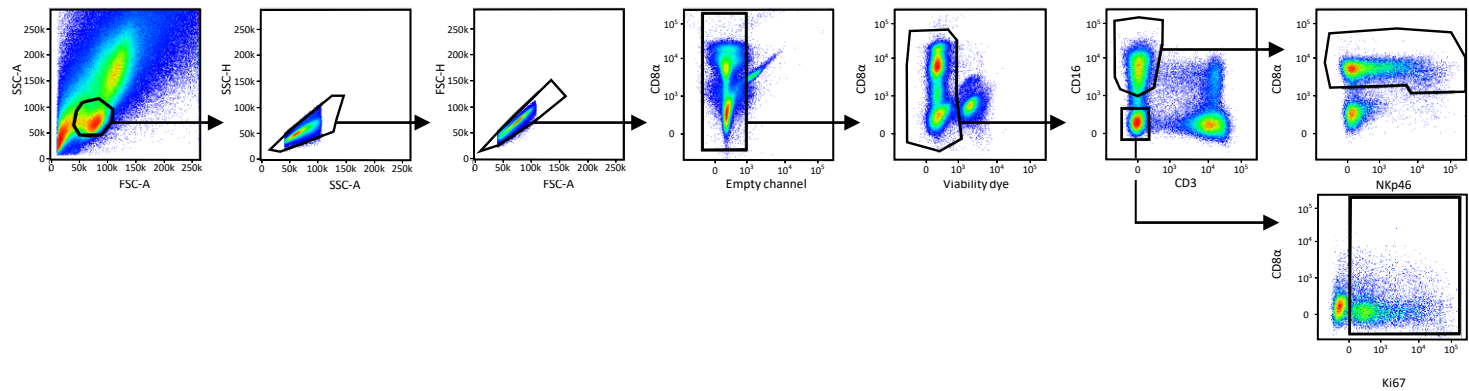

Supplement: Supplementary file 1 — Additional file 1 Gating strategy for identifying porcine NK cells. A Gating strategy for maternal blood samples, B maternal endometrium, and C fetal placenta tissue samples. For each of the three staining panels used in this study, lymphocytes were selected according to their light scattering properties (SSC-A against FSC-A). This was followed by a two-step doublet discrimination (SSC-H against SSC-A, and FSC-H against FSC-A), and subsequently CD8α was gated first against an empty channel to eliminate autofluorescence in the sample, and then against a viability dye. NK cells were then specifically selected by gating on CD16+/CD3- cells, followed by selecting all cells which displayed a CD8α+/NKp46- or NKp46+ phenotype as well as those displaying a CD8αdim/NKp46high phenotype. The expression threshold between Ki-67+ and Ki-67- cells within the NK cells was often difficult to determine. We therefore set the gate for Ki-67+ according to a group of non-NK cells showing a CD16-/CD3- phenotype which showed a clear +/− separation for this marker. The representative samples presented here were from freshly isolated, unstimulated phenotyping samples. [file 13567_2025_1623_MOESM1_ESM.pdf]

NK cells within total lymphocytes

Control

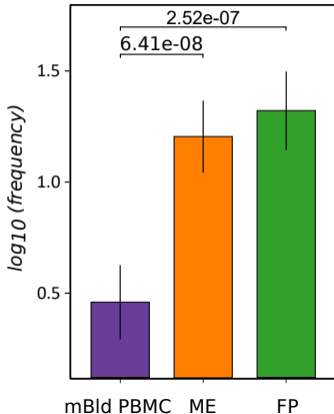

Infected

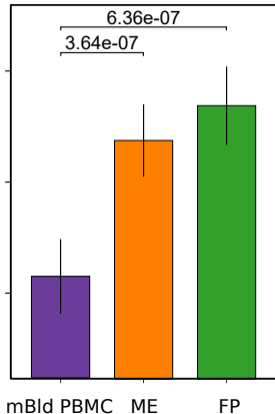

Supplement: Supplementary file 2 — Additional file 2 NK cell population within total lymphocyte population. A linear mixed effects model considering infection status, tissue, and the interaction between both was applied. A random intercept (gilt) was fitted and estimated marginal means (emmeans) were calculated using measure population frequencies. The y-axis depicts the population of NK cells relative to total lymphocytes from each respective tissue group in a log10 transformed scale. Only significant p-values are shown (P 0.1). The whiskers depict the 95% conﬁdence intervals of the emmeans. [file 13567_2025_1623_MOESM2_ESM.pdf]

PBMC

ME

FP

Total NK

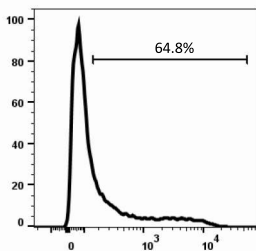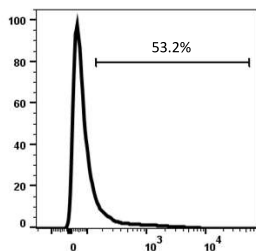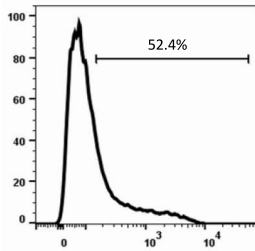NKp46<sup>-</sup>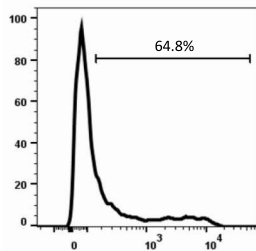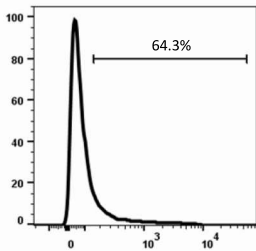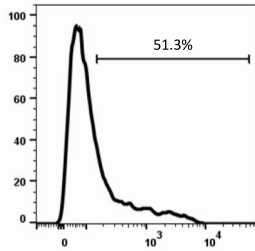NKp46<sup>+</sup>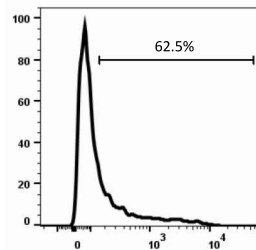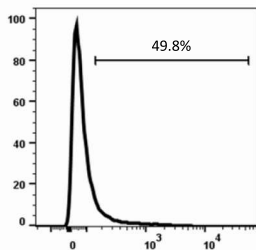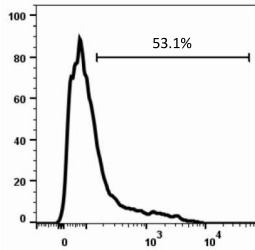NKp46<sup>High</sup>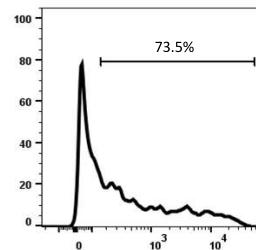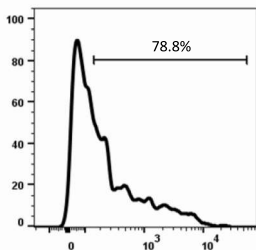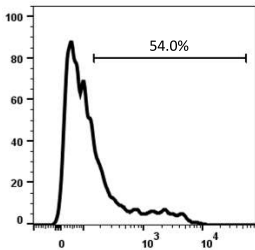

Ki-67

Supplement: Supplementary file 3 — Additional file 3 Representative gating for Ki-67 expression within total NK cells and NKp46-defined NKsubpopulations. The percentage of positive cells from the sample total is given above the line representing the zoneof positive expression. [file 13567_2025_1623_MOESM3_ESM.pdf]

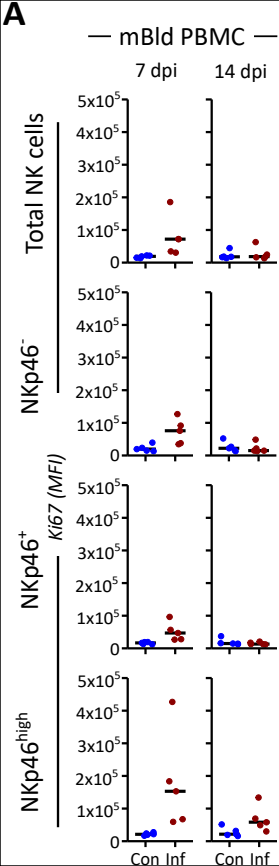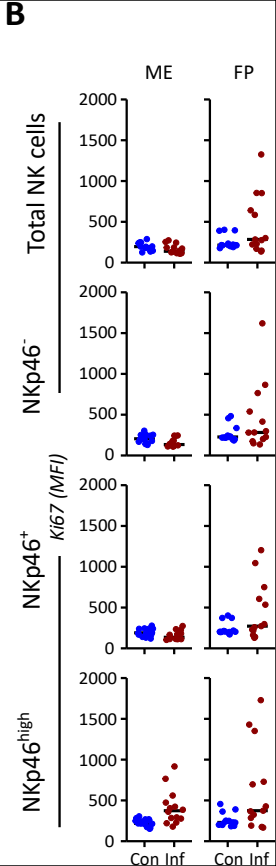

Supplement: Supplementary file 4 — Additional file 4 MFI of Ki67 expression. A MFI of Ki67 from maternal PBMCs from days 7 dpi and 14 dpi. The median value of each subgroup is depicted with a black bar. B MFI of Ki67 from putative uNK cells isolated on day of necropsy from ME and FP tissue samples. The median value of each subgroup is depicted with a black bar. [file 13567_2025_1623_MOESM4_ESM.pdf]

mBld PBMC

ME

FP

Total NK

NKp46<sup>-</sup>NKp46<sup>+</sup>NKp46<sup>high</sup>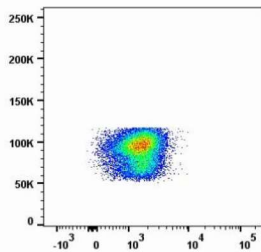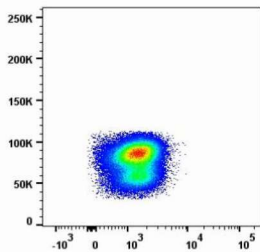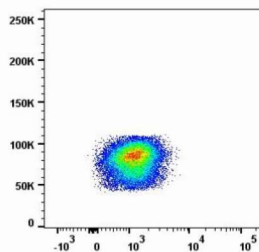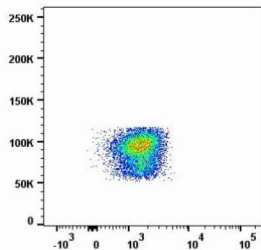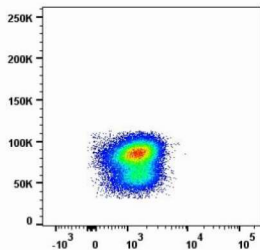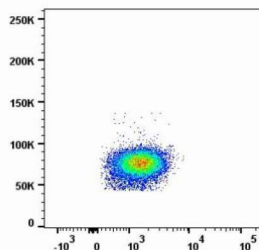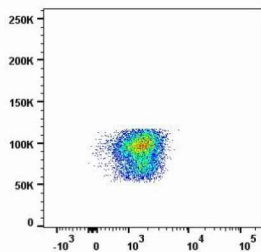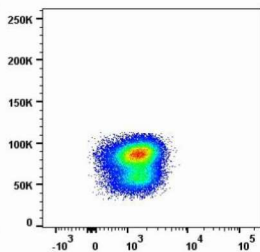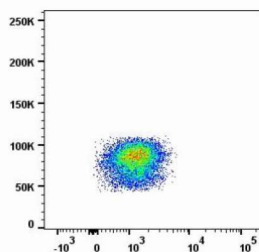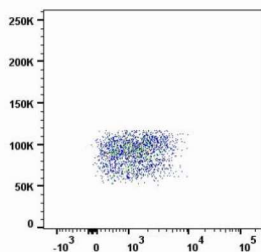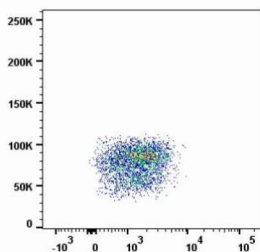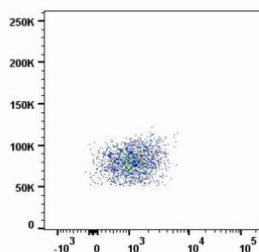

Pertorin

Supplement: Supplementary file 5 — Additional file 5 Dot plots of perforin expression frequency within NK cells. Dot plots demonstrating the universal expression of perforin within the total NK cell population and NKp46-defined subpopulations of all three tissue types measured. Y-axis is forward scatter area (FSC-A). [file 13567_2025_1623_MOESM5_ESM.pdf]

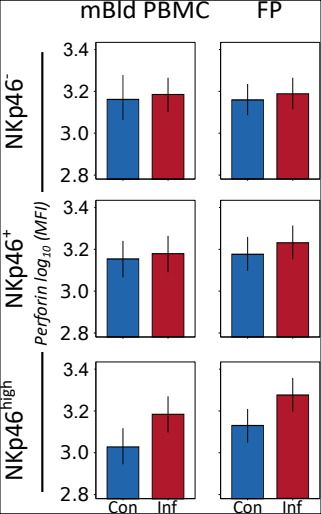

Supplement: Supplementary file 6 — Additional file 6 MFI of perforin expressed in FP and maternal PBMCs isolated on day of necropsy, grouped into NKp46-defined subpopulations. A linear mixed effects model considering infection status, tissue, and the interaction between both was applied. A random intercept (gilt) was fitted and estimated marginal means (emmeans) were calculated using measured population perforin MFI values. The y-axis depicts the population of the NKp46-defined NK cell subpopulation relative to total NK cells from each respective tissue group and infection status in a log10 transformed scale. The whiskers depict the 95% conﬁdence intervals of the emmeans. [file 13567_2025_1623_MOESM6_ESM.pdf]

mBld PBMC

ME

FP

Total NK cells

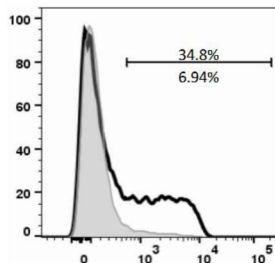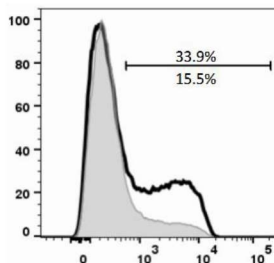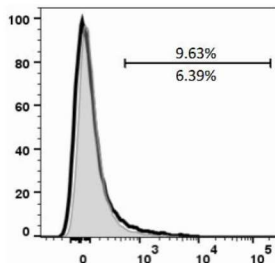

Nkp46

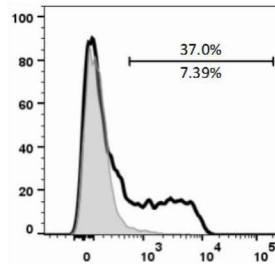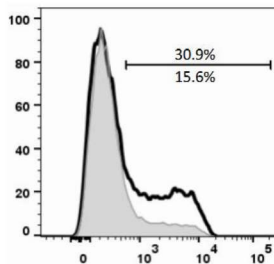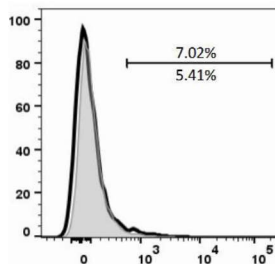Nkp46<sup>+</sup>/high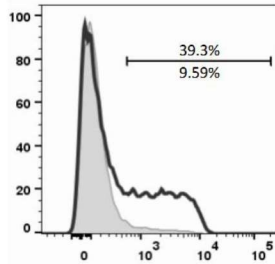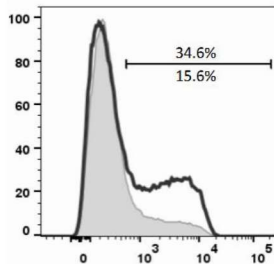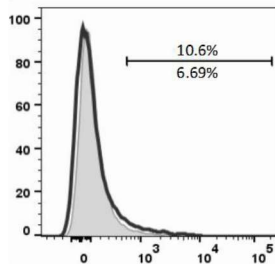

CD107a

Supplement: Supplementary file 7 — Additional file 7 Representative gating for CD107a expression within total NK cells and NKp46-defined NK subpopulations. The grey shaded area represents the sample with no K562 target cells added, and the unshaded area with the thick outline represents the sample with the K562 target cells added in a target cell to effect cell ratio of 10 to 1. The value above the gating line is the value recorded from the sample with target cells, and the value below the line is the value recorded from the control sample without target cells. [file 13567_2025_1623_MOESM7_ESM.pdf]

mBld PBMC

ME

FP

Total NK cells

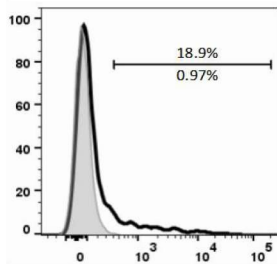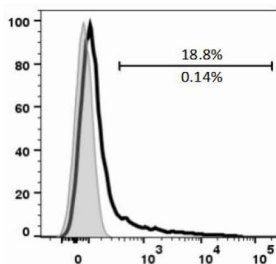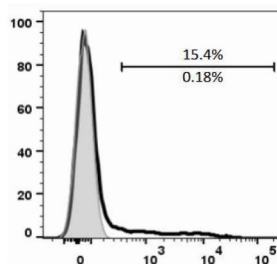NKp46<sup>-</sup>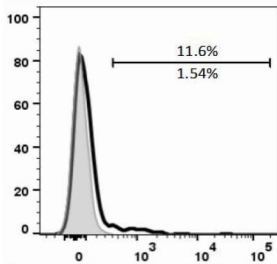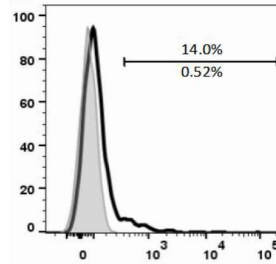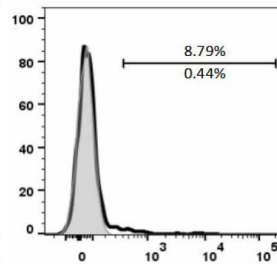NKp46<sup>+/high</sup>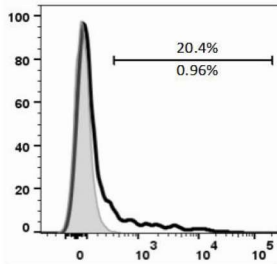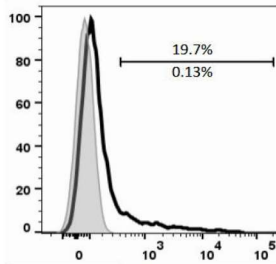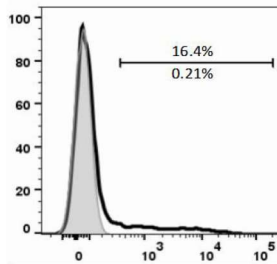IFN $\gamma$

Supplement: Supplementary file 8 — Additional file 8 Representative gating for IFNγ expression within total NK cells and NKp46-defined NK subpopulations. The grey shaded area represents the unstimulated sample incubated in only cell culture medium, and the unshaded area with the dark outline represents the sample incubated in a cytokine stimulation mixture of IL-2, IL-15, and IL-18. The value above the line represents the value recorded from the stimulated sample, and the value below the line represents the value recorded from the unstimulated sample. [file 13567_2025_1623_MOESM8_ESM.pdf]
